# A Pilot Study of a CustomGPT for the Royal College of Ophthalmologists Curriculum 2024

**DOI:** 10.1177/23821205261474025

**Published:** 2026-09-11

**Authors:** William Purcell, Vikas Chadha, Devina Gogi, Vernon Long, Aabgina Shafi, Yashin Ramkissoon

**Affiliations:** 1 6979 Department of Ophthalmology, Sheffield Teaching Hospitals NHS Foundation Trust, Sheffield, United Kingdom; 2 565605 Tennent Institute of Ophthalmology, Gartnavel General Hospital, Glasgow, Scotland, United Kingdom; 3 4472 Department of Ophthalmology, Leeds Teaching Hospitals NHS Trust, Leeds, United Kingdom; 4 156677 Department of Ophthalmology, Pinderfields Hospital, Wakefield, United Kingdom

**Keywords:** Large language model, Royal College of Ophthalmologists, Ophthalmic Specialist Training

## Abstract

**Background:**

Large language models (LLMs) employ transformer architectures to generate contextually appropriate responses and are increasingly applied in medical education. To support implementation of the Royal College of Ophthalmologists (RCOphth) Ophthalmic Specialist Training (OST) Curriculum 2024, the Yorkshire & Humber School of Ophthalmology developed a CustomGPT trained exclusively on official RCOphth curriculum documents. This study evaluated its usability, effectiveness, and accuracy.

**Methods:**

A pilot study invited ophthalmology trainees (ST1–ST7, Fellows) and trainers (Consultants, Supervisors, Tutors, Programme Directors) to use the CustomGPT over a three-month period (April to June 2025). Quantitative feedback (Likert scales) assessed relevance, ease of use, and satisfaction, while qualitative feedback explored perceived value and limitations. Accuracy was evaluated by comparing responses from the CustomGPT and the RCOphth Training Committee (TC) Chair to ten authentic curriculum queries.

**Results:**

Twenty-eight participants (14 trainees, 14 trainers) completed the survey; only 21% were regular AI users. Mean scores were high for relevance (4.21/5), ease of use (4.38/5), and satisfaction (4.10/5), with 86% reporting time savings and 75% rating their likelihood of recommending the tool as 8/10 or higher. Qualitative data highlighted concise, accurate, and well-structured responses but noted occasional technical issues and rigid interpretation of complex queries. Comparative analysis showed strong alignment with the TC Chair, though the GPT sometimes demonstrated greater precision in referencing but less flexibility in interpretation.

**Conclusions:**

A curriculum-specific GPT can provide rapid, accurate guidance, reducing ambiguity and improving efficiency. When integrated into training platforms, such tools can enhance curriculum accessibility in postgraduate medical education.

## Introduction

Large language models (LLMs) use deep learning techniques, particularly transformer architectures, to learn patterns in language and generate contextually appropriate responses to user queries.^1^ Earlier natural language processing systems relied on rules or models such as recurrent neural networks, which often lost context in longer texts.^2^ Transformers process all words in a text simultaneously rather than sequentially, allowing LLMs to maintain context over long passages and making them a major step forward in handling complex language.^1^

These advances have enabled LLM’s to demonstrate strong performance across a range of knowledge intensive tasks including summarisation and question answering; they are being increasingly explored for clinical and education applications in medicine.^3,4^

LLMs are increasingly used in medical education, with evidence suggesting they enhance learning by synthesising information, supporting personalised learning, and improving accessibility.^5^ Several studies have demonstrated that LLM’s can perform at or near the level of medical trainees on professional examinations, highlighting their potential role as educational tools.^4^ Within ophthalmology, recent research has evaluated the performance of LLM’s in answering ophthalmology examination questions and generating clinical information, with generally promising results but concerns regarding factual accuracy and hallucinations.^6-8^ To improve reliability, researchers have begun developing domain specific implementations of LLM’s configured using speciality specific knowledge sources.^9^ In ophthalmology, specialised LLM’s have been explored for patient education and trainee learning, demonstrating potential improvements in accuracy and usability compared to general purpose LLM’s.^9,10^

The Royal College of Ophthalmologists (RCOphth) launched its new Ophthalmic Specialist Training (OST) Curriculum 2024 in August 2024,^11^ marking a significant evolution from the previous 2010 curriculum. This updated curriculum is fundamentally competency-based rather than time or numbers based, emphasising the quality of evidence over quantity. It is structured around seven domains of professional skills and knowledge, which are mapped to the General Medical Council’s (GMC) Generic Professional Capabilities (GPCs) framework. These domains include Patient Management, Health Promotion, Leadership and Team Working, Patient Safety and Quality Improvement, Safeguarding and Holistic Patient Care, Education and Training, and Research and Scholarship. The Patient Management domain is further divided into twelve Special Interest Areas (SIAs).

Progression through training is defined by four critical advancement points, corresponding to Levels 1, 2, 3, and 4. Each level has high-level, overarching learning outcomes, supported by detailed descriptors in supplementary syllabi. The assessment strategy, outlined in the “Assessment Strategy” and “Curriculum Handbook”^11^ employs new tools such as Entrustable Professional Activities (EPAs) for the Patient Management domain and Generic Skills Assessment Tools (GSATs) for the other six generic domains. Other established assessment tools, including Multi-Assessor Reports (MARs), Multi-Source Feedback (MSF), Objective Assessment of Surgical and Technical Skills (OSATS), Direct Observation of Procedural Skills (DOPS), Case-based Discussions (CbDs), and audits, have been adapted and retained. These assessments provide both formative feedback and summative judgements for progression decisions.

To support trainees and trainers in navigating this new, complex curriculum, a CustomGPT was developed by the Yorkshire & Humber School of Ophthalmology. This system was configured exclusively on the full complement of official RCOphth Curriculum 2024 documents.^11^ Its primary objective is to deliver rapid, concise, and unambiguous responses to curriculum enquiries. We then conducted a pilot study to evaluate its usability, effectiveness and accuracy including a comparative analysis of the CustomGPT system’s responses against those of the RCOphth Training Committee Chair (human expert) for a series of authentic curriculum-related questions.

## Methods

### GPT Design and Configuration

A CustomGPT was developed on OpenAI’s platform using GPT-4o between March and April 2025. The system was configured using the OpenAI CustomGPT framework to operate exclusively on the approved RCOphth OST Curriculum 2024 documents. Source materials included the full curriculum syllabi, Assessment Strategy, Assessment Blueprint, Curriculum Handbook, and Matrix of Progression.^11^ All documents were uploaded directly to the model’s knowledge base in PDF format, enabling retrieval augmented generation (RAG) from embedded sources. This constrained the model’s knowledge environment to the official curriculum during inference. External browsing and web search capabilities were also explicitly disabled, preventing the model from incorporating external information during the inference process. These restrictions were implemented to minimise the risk of hallucinations and maintain precise answers extracted from the underlying knowledge base.

During configuration, the model was provided with a structured system prompt designed to constrain it’s operational scope and ensure response alignment. The prompt instructed the model to respond only to questions related to the OST curriculum structure, learning outcomes, progression requirements, assessment methods and portfolio evidence recommendations. Additional prompt constraints further required the model to verify source information by directly extracting content from uploaded PDF documents before generating responses. Response behaviour was further standardised through formatting and safety rules. Outputs were limited to concise responses of approximately 100-200 words using terminology appropriate for NHS training contexts. The model was also instructed to avoid speculation or extrapolation beyond the uploaded curriculum documents. This prompt engineering strategy ensured that responses remained tightly aligned with the official OST curriculum while reducing the likelihood of unsupported or hallucinated outputs.

### Survey Design

#### Participant Inclusion and Exclusion Criteria

Ophthalmology trainee participants included a broad representation of the training pathway from Specialty Trainees (ST) year 1 (ST1) to year 7 (ST7), as well as post-CCT Fellows, ensuring representation from junior through senior stages of training. Eligible trainers included Consultant Ophthalmologists, Educational Supervisors, Named Clinical Supervisors, College Tutors, and Training Programme Directors. In addition, key stakeholders were invited, such as members of the Royal College of Ophthalmologists Training Committee and representatives from the Yorkshire and Humber School of Ophthalmology, including the Head of School and a trainee representative. No specific stakeholders were excluded as participants, although the model and survey was only distributed to the described trainees and trainers.

#### Recruitment

Formal invitations were distributed via email to a cohort of 120 potential respondents (trainers and trainees) in the Yorkshire and Humber School of Ophthalmology. Each invitation detailed the purpose and expected duration of the study and provided a direct link to the CustomGPT along with clear user instructions. Users were instructed to avoid entering personally identifiable information such as patient or colleague names. A link to the OpenAI privacy policy was also included. Data over a 3-month period from April 2025 to June 2025 was collected for analysis.

#### Collecting User Feedback

The survey was carried out using Microsoft Forms (Microsoft Corporation, Redmond, WA, USA) and included both quantitative and qualitative components (Supplementary file 1). Quantitative feedback was obtained using structured questions with Likert-type rating scales assessing relevance, ease of use and overall satisfaction. Qualitative feedback was collected through open-ended questions designed to gather insights, highlight strengths and limitations, and provide suggestions for future improvement.

### Comparative Analysis: CustomGPT Versus RCOphth Training Committee Chair

To validate the accuracy of the CustomGPT’s responses, we compared it against the RCOphth Training Committee (TC) Chair, a human expert involved in the development of the curriculum. Ten questions based on real-world curriculum scenarios were submitted to both the CustomGPT and the TC Chair. Each response was then compared and assessed for accuracy, detail, and adherence to the official RCOphth Curriculum 2024 guidance.

### Study Ethics

The study followed the Declaration of Helsinki’s principles. Participants were instructed to avoid entering personal information into the CustomGPT system. Data management complied with OpenAI’s privacy policy, and conversation records were not used for training OpenAI models. The reporting of this study conforms to the strengthening the reporting of observational studies in epidemiology (STROBE) statement^12^ (Supplementary file 2).

## Results

### Survey Results

The study was sent to 120 potential respondents. A total of 28 of those completed the survey, comprising 14 Consultants and 14 STs or Fellows. This represented a broad range of training levels. Of these, only 21% (6/28) reported being regular users of Artificial Intelligence tools.

The GPT’s response relevance received a mean rating of 4.21/5. Ease of use was rated highly, with a mean score of 4.38/5, while overall satisfaction with the system achieved a mean score of 4.10/5. Respondents rated the likelihood of recommending the tool to other trainees or supervisors with a mean score of 7.9/10. A total of 21/28 (75%) participants gave a score of 8/10 or higher. This indicates that a large majority were very satisfied with this approach to answering curriculum queries. 24/28 (86%) of users felt the system saved them time compared to traditional methods of curriculum query support.

Qualitative feedback highlighted that the tool provided quick answers and was easy to use. Users reported detailed responses of curriculum requirements with practical application to their queries (Table 1). Limitations included technical issues and difficulties handling some specific or complex queries, while some users also felt that the system only had limited added benefit compared to openly available commercial LLMs. Suggested improvements included integration of the CustomGPT into the RCOphth website and the new trainee e-portfolio.

**Table 1.** Examples of Qualitative Feedback on Strengths, Limitations and Suggestions for Future Improvement Reflecting the Themes Identified (Verbatim Transcript)

What aspects of the system worked well?	What aspects of the system did not work well?	What additional features would improve this custom GPT curriculum guide?
I had certain questions related to the numbers of different forms I should submit, and this chat gave me a very detailed answer, even more than what I expected	Sometimes the system is not able to answer specifics. The system loads the code at times rather than the answer	After a short trial period, it is an extraordinary and very helpful tool. I may suggest changing the background of the chat to reflect its purpose
Easy access summary of all the different forms/assessment tools/abbreviations. Good summary of what residents require for ARCP.	On a couple of occasions got search code but after a while the answer appeared	Options with links where expansion of a certain topic could be obtained
Bullet point answer - easily read and easy comprehension	I wanted specific examples of evidence for the different levels to suggest the residents undertake e.g. Neuro-ophth visual fields, examples of evidence for each level	If it could help with fourteen fish!! (The new e-portfolio system)
Helps to find answer much quicker than traditional method of looking through long pdf files. Also help to compare and contrast competencies required at different levels of specific SIA in table form (e.g. VR, cataract)	Has just not worked on a couple of occasions- can’t really understand what it offers which is better than using chat GPT normally	There is a limit of asking questions in ChatGPT and had to wait for 5 hours before I can ask GPT again, unless upgrading to ChatGPT plus. If we could ask unlimited questions that would be much more helpful, rather than having to wait for few hours before trying again.
Comprehensive answers, relevant resources provided and good signposting. Aware of the criteria set out by RCOphth on assessments and requirements in training.	I typed the same question in my web browser and got similar answers. If this customisation ChatGPT is incorporated into the training website it can be useful while on it. Outside that any AI would answer in similar manner?	Can’t think of any
It was easy to use. When asked about EPA it gave a detailed answer including the forms used, how to complete and by whom.	It didn’t quite answer the question asked as we’d already tried all the suggestions but was helpful that we were on the right track	If it is quicker

### Comparative Analysis: Training Committee Chair vs. Custom GPT

The comparative analysis of the custom GPT’s responses against those of the RCOphth TC Chair for the ten curriculum queries is summarised in Table 2.

**Table 2.** Comparative Analysis of the Custom GPT’s Responses Against Those of the RCOphth TC Chair for the Ten Curriculum Queries

Theme	Query	GPT response summary	Human expert response summary	Curriculum alignment & analysis
1. Level 1 Cataract competencies	What cataract surgery competencies are required to be signed off for Level 1?	Comprehensive and accurate list of competencies which also specifies required EPA, MSF and MAR.	Accurate summary of competencies. Focused on the minimum competencies for Level 1 cataract surgery.	GPT and Human responses were comparable in content and factually correct but the GPT provided more detailed explanation of each requirement with examples of clinical activity that could be evidenced.
2. In-progress leadership activities for Level 1 GSAT sign-off (ST1)	I am an ST1 working at Level 1. I have started some leadership activities, but they will not be finished before the ARCP. Can I include them as a competency in my GSAT form to get Level 1 signed off?	Advised that in-progress activities could be included, emphasizing initiative, insight, and intent, stating it is “usually sufficient” for Level 1 sign-off if other evidence is strong.	Stated that if all competencies for all six generic domains are not acquired, the Level 1 GSAT would not be signed off as ‘achieved’. Advised to continue adding competencies in subsequent rotations until all are acquired.	Human response was more accurate regarding the summative nature of GSAT sign-off for level progression. The curriculum requires achieving “all the Learning Outcomes in all domains in the current level” for advancement. While documenting in-progress work is valuable formatively, the GPT’s assertion of “usually sufficient” for summative sign-off is inaccurate if all six general domains are not completed.
3. Mandatory Level 4 Cataract competencies	What competencies are mandatory to achieve Level 4 in Cataract Surgery?	Provided a highly detailed and comprehensive list including independent performance of complex cataract surgeries (specific types like high myopia, pseudoexfoliation, small pupils, post-trauma), use of various intraocular lenses (IOLs), management of complications (capsule tears, iris trauma, vitrectomy), independent clinic management, Level 3/4 supervision, continuous audit, a personal audit of 50 cases with refractive data, EPA Level 4 for Cataract Surgery and for managing an operating list, OSATS1 for complex surgery, consultant observation, CbDs, MARs, and logbook reviews.	Provided a detailed list, including diagnosing/managing cataract in all settings, independent clinic management, performing all levels of complex surgery independently, managing intraoperative complications (capsular tears, vitreous loss, sulcus IOL), supervising/signing off residents to Level 3, and using monofocal, toric, and anterior chamber lenses.	Both responses were accurate. However, the GPT was more comprehensive, with increased granularity of descriptors within level 4 curriculum.
4. Mandatory DOPS in Urgent Eye Care	Are there any mandatory DOPS I need to do in Urgent Eye Care?	Stated “Yes” and listed Corneal gluing and Vitreous biopsy as mandatory skills that could be evidenced by DOPS or other observation methods, particularly from Level 3 onwards. Also mentioned OSATS requirements for microsurgical skills, trauma, and lateral cantholysis.	Stated there are no mandatory DOPS in Urgent Eye Care at any Level.	The GPT and human response were contradictory. Although the human response was technically correct that are no mandatory DOPS specific to the location of Urgent Eye Care. The GPT reflected curriculum documents which list specific procedures that can be evidenced by DOPS as mandatory requirements for the Urgent Eye Care EPA.
5. Number of cataracts for Level 2 training	How many cataracts do I need to perform to achieve level 2 training?	Explicitly stated “no specific numerical requirement”, highlighting the curriculum’s shift away from fixed numbers. Emphasised holistic assessment through logbook, audit, Refraction Certificate, EPA, MSF, and ARCP outcome.	Explicitly stated “no mandatory number of cataracts” and reinforced the need to demonstrate acquired competencies.	Both responses were accurate and consistent with the curriculum’s philosophy that training is competency-based, not numbers-based.
6. Achieving Level 3 in Community Ophthalmology	How do I achieve level 3 in community ophthalmology?	Provided a detailed explanation: complete Level 3 EPA for Community Ophthalmology (demonstrating independent assessment, management of moderate complexity patients, safe decision-making, evidence application, appropriate investigations/referrals). Also listed relevant evidence types (CbDs, direct observations, MARs, reflections, teaching, audit activities) and the need for NCS sign-off.	Stated that the resident needs to demonstrate acquisition of “all the competencies laid out in the syllabus for that specific level three SIA”.	While the human response was correct, the GPT provided a significantly more comprehensive and actionable answer, detailing the primary assessment tool (EPA Level 3 Community Ophthalmology) and specific categories of evidence required, aligning with the curriculum.
7. Research requirement for Level 3 training	Do I need research to achieve level 3 training?	Stated “Yes,” as Research and Scholarship is a curriculum domain, requiring competence in understanding principles, critical appraisal, informing clinical practice, and participating in audit/QI. Clarified that original research is not mandatory but engagement is essential.	Stated “Yes,” identifying Research and Scholarship as a generic domain, requiring acquisition of all Level 3 competencies in this and other domains.	Both responses were accurate and consistent. The GPT added slightly more informative detail about the specific elements of “competence in Research and Scholarship” at Level 3.
8. OST4 requirements to achieve Level 3	I am an OST4 doctor. What do I need to achieve level 3?	Provided an extensive and precise list including: EPA Level 3 for each of the 12 SIAs (minimum one every 6 months), Part 2 FRCOphth (Written and Oral), up-to-date ePortfolio, MSF every 12 months, ESR every 6 months, surgical logbook (A/PS/P), cataract complications audit, MARs, GSATs for non-clinical domains, and mandatory WpBAs (CRS, DOPSBi, CbDs, OSATS).	Stated the need to acquire competencies in all seven domains (Patient Management with 12 SIAs, and six generic domains) and pass the FRCOphth Part 2 Oral examination.	The human response was correct, but the GPT provided a more comprehensive and practically useful summary. The GPT was acccurate in detailing the numerous specific assessment tools, examinations, and audit requirements outlined in the “Curriculum Handbook” and the “ARCP Matrix of Progression”.
9. Sign-off for Level 1 Cataract despite surgical struggles (no full case)	Question from one of my college tutors - if a trainee is really struggling with cataract surgery (still not completed a full case) and in experienced supervisors judgement slower to acquire skills having applied self diligently and reflected, has done individual steps, would they be signed off for Level 1? The trainee says they should be signed off as no numbers are required. ES is happy to sign off all other areas, but has reservations about intra-ocular surgical skills.	Stated that sign-off for Level 1 is possible if other Level 1 Learning Outcomes are met, re-emphasising the competency-based approach. Listed relevant evidence: individual steps via OSATS/direct observation, operating microscope, logbook (assisted/supervised), simulation, reflection, MARs, audit. Suggested recording reservations and planning targeted support.	Provided a highly precise and critical response: “Level one in cataract surgery only requires the training to be able to demonstrate the application of aseptic technique and the competent use of the operating microscope. Therefore the supervisor can sign off this trainee at level one. Further development of the intraocular surgical skills will be assessed in the subsequent levels”. This directly cites Level 1 descriptors for cataract.	While the GPT correctly articulated the competency-based principle, the human expert offered a more insightful and precise answer by pinpointing the minimal, specific Level 1 requirements for cataract surgery, making sign-off viable despite broader surgical struggles. The human response effectively demonstrated expert interpretation of the curriculum’s specific expectations for that level and SIA.
10. Completing Level 3 SIAs before completing Level 2?	I am a resident working at level 2. Can I complete SIAs in Level 3 before completing Level 2?	Stated a rigid “No,” arguing that “Level 2 must be fully signed off before you are eligible to begin Level 3 SIAs,” citing sequential progression.	Provided a nuanced “Yes,” explaining that while formal completion/sign-off is sequential (“you can still acquire the competencies of the various special interest areas in level three... at the time of completion of level two”), this requires ES confirmation and ARCP panel ratification.	The human response was more accurate in reflecting the curriculum’s inherent flexibility. The curriculum allows for “spiral learning” and “simultaneous acquisition” of competencies, particularly for SIAs like Community Ophthalmology and Urgent Eye Care. The GPT adopted a more rigid interpretation, overlooking the distinction between competency acquisition and formal sequential sign-off for progression.

## Discussion

The RCOphth Curriculum 2024 presents challenges in both scale and complexity, with trainees and trainers frequently required to cross-reference multiple documents to clarify requirements. To address this, we developed a CustomGPT configured exclusively on the official curriculum to provide accurate and accessible responses. In our pilot study, the system demonstrated an ability to provide fast and relevant responses in a user-friendly manner.

### Survey Feedback

Despite limited prior experience with AI, participants consistently reported that the tool was intuitive, providing detailed, well-structured, and relevant answers. User ratings for relevance (4.21/5), ease of use (4.38/5), and overall satisfaction (4.1/5) were encouraging, and three-quarters of respondents indicated a strong likelihood of recommending the tool to others. Many noted substantial time savings compared with manually searching PDFs. Qualitative feedback highlighted the comprehensiveness of answers, effective signposting, and helpful links to resources, though improvements were suggested in addressing technical issues and enhancing responses to complex or highly specific queries.

A minority of users perceived only modest additional value compared with open-access LLMs. Although publicly available models can produce satisfactory responses across a wide range of curriculum-related queries, evidence indicates that LLMs trained with supplementary proprietary data may achieve greater accuracy in specific domains.^3^ Restricting responses to defined source materials is particularly advantageous given the specialised and continually evolving nature of medical curricula. While outputs may appear similar to those from open-access models, the key distinction lies in their traceability, controlled scope, and alignment with curriculum content. These factors help mitigate the risks of hallucination, inappropriate guidance, and inclusion of outdated or non-UK-specific material. Despite the relatively small number of respondents, the feedback provides an encouraging indication of the CustomGPT’s operational advantage in overcoming the physical navigation challenge presented by extensive curriculum documentation.

### Comparative Analysis: Training Committee Chair Versus Custom GPT

Comparison with responses from the TC Chair demonstrated both alignment and divergence. In most cases, the CustomGPT provided information consistent with the official curriculum, often with greater detail and structure than the human expert. A significant discrepancy emerged around mandatory Direct Observation of Procedural Skills (DOPS) in Urgent Eye Care. While the Chair asserted that no mandatory DOPS exist, the GPT recognised that certain procedures are mandatory and can be evidenced by DOPS among other methods. Reference to a curriculum footnote confirmed the AI’s interpretation, suggesting that a strictly source-grounded approach may sometimes reduce the risk of oversimplification or recall error inherent in human responses. Conversely, when asked about progression through training levels, the GPT applied the curriculum’s structure rigidly, stating that Level 3 Special Interest Areas cannot be undertaken until Level 2 is complete. The human expert, however, described a more pragmatic reality in which Level 3 competencies may be developed earlier, subject to later ratification in that specific Level 3 EPA. This illustrates both the strength and the limitation of the CustomGPT: while exhaustive in its use of documentation, it may struggle to capture the intended flexibility and professional judgement embedded within a competency-based training model.

It is also important to recognise that, in typical educational settings, supervisors may not possess the same depth of expertise as College Curriculum Chairs; therefore, in real-world use, the relative advantage of the CustomGPT over human-responses may be even more pronounced.

## Limitations

This study has several limitations. The sample size was small (n=28), limiting statistical power and the ability to draw generalisable conclusions; the findings should be considered exploratory. As with all LLM’s, the CustomGPT system can be subject to limitations such as hallucinations, prompt misinterpretation, or incomplete retrieval of information, despite being configured with specific curriculum documents. In addition, the long-term reliability of such systems may be affected by updates to underlying AI models or curriculum changes, requiring ongoing monitoring and validation. The analysis relied primarily on descriptive statistics due to the small cohort size and there was potential for selection bias as those who chose to respond may have been inherently more interested in AI tools. Larger national studies with broader recruitment would be required to minimise selection bias and confirm findings with more robust statistical analysis.

## Conclusion

Taken together, these findings suggest that a curriculum-specific GPT can function as a highly effective supplementary guidance system. Its ability to synthesise information from multiple documents, present it with clarity, and remain aligned with official sources offers trainees and trainers a reliable means of navigating an otherwise complex framework. While not a replacement for expert interpretation, the tool may reduce ambiguity, improve efficiency, and allow human experts to focus on higher-order educational discussions rather than routine clarification of documentation. Future refinement should aim to strengthen the model’s handling of nuance, particularly where curriculum philosophy relies on flexibility, and to embed the tool within existing platforms such as the trainee e-portfolio to maximise utility and adoption. In addition, further validation in larger, nationally representative cohorts will be important to confirm findings and ensure the ongoing reliability of the tool as both AI systems and training curricula evolve.

Most importantly, this study illustrates a concept that extends well beyond Ophthalmology: domain-specific GPTs trained solely on authoritative documents can enhance the clarity, accessibility, and reliability of complex professional curricula. Such systems have the potential to transform postgraduate medical education across specialties, ensuring that trainees and trainers spend less time navigating documentation and more time focused on meaningful clinical learning.

## Supplemental Material

Supplemental Material - A Pilot Study of a CustomGPT for the Royal College of Ophthalmologists Curriculum 2024 Supplemental Material for A Pilot Study of a CustomGPT for the Royal College of Ophthalmologists Curriculum 2024 by William Purcell, Vikas Chadha, Devina Gogi, Vernon Long, Aabgina Shafi, Yashin Ramkissoon in Journal of Medical Education and Curricular Development

Supplemental Material - A Pilot Study of a CustomGPT for the Royal College of Ophthalmologists Curriculum 2024 Supplemental Material for A Pilot Study of a CustomGPT for the Royal College of Ophthalmologists Curriculum 2024 by William Purcell, Vikas Chadha, Devina Gogi, Vernon Long, Aabgina Shafi, Yashin Ramkissoon in Journal of Medical Education and Curricular Development

## Appendix

### Abbrevations

LLM: Large language model
RCOphth: Royal College of Ophthalmologists
OST: Ophthalmic Specialist Training
