# Supplementary material for: A Pilot Study of a CustomGPT for the Royal College of Ophthalmologists Curriculum 2024
Source: J Med Educ Curric Dev. 2026 Sep 11;13:23821205261474025. doi: 10.1177/23821205261474025 (PMC13570001; doi:10.1177/23821205261474025)
Supplement: Supplementary file 1 [file sj-pdf-1-mde-10.1177_23821205261474025.pdf]

Survey Questionnaire

Questionnaire title:
OST Curriculum 2024 Custom GPT Guide – User Feedback

Questionnaire introduction shown to participants:
Feedback survey on the usability and performance of the custom GPT designed to support residents and trainers navigating the new curriculum.

Note:
This questionnaire was administered via Microsoft Forms. No participant response data are included in this file.

Survey Questions

1. Role

Response type: Single choice
Required: Yes

Please select your role.

- ST1
- ST2
- ST3
- ST4
- ST5
- ST6
- ST7
- Consultant
- Educational supervisor
- Named clinical supervisor
- College tutor
- Other

---

2. Level of training

Response type: Single choice
Required: Yes

Please select your level of training.

- Level 1
- Level 2

- Level 3
- Level 4
- Not applicable
- Other

---

3. How often do you use AI?

Response type: Single choice
Required: Yes

- Regular user
- Occasional user
- Non user

---

4. Rating this system

Response type: Rating matrix
Required: Yes

Participants were asked to rate the following items on a 5-point scale:

Scale:
1 = Poor
2 =
3 =
4 =
5 = Excellent

Item	1	2	3	4	5
How relevant were the answers to your query?	☐	☐	☐	☐	☐
How easy was it to use the system?	☐	☐	☐	☐	☐
Overall satisfaction with the system?	☐	☐	☐	☐	☐

---

5. Did this system save you time compared to traditional methods?

Response type: Single choice
Required: Yes

Additional explanatory text shown to participants:
In comparison to how you would usually obtain curriculum and portfolio related information.

- Yes
- No
- Unsure

---

6. How likely are you to recommend this custom GPT guide to other residents or supervisors?

Response type: 11-point rating scale
Required: Yes

Scale:
0 = Not at all likely
10 = Extremely likely

0 1 2 3 4 5 6 7 8 9 10
☐ ☐ ☐ ☐ ☐ ☐ ☐ ☐ ☐ ☐ ☐

---

7. What aspects of the system worked well?

Response type: Free-text response
Required: Yes

Prompt shown to participants:
For example, state how the system helped resolve your training queries.

Response:

---

8. What aspects of the system did not work well?

Response type: Free-text response
Required: Yes

Prompt shown to participants:
For example, state if a query was not resolved or if you were aware of any inaccurate or misleading answers.

Response:

---

9. What additional features would improve this custom GPT Curriculum guide?

Response type: Free-text response
Required: Yes

Prompt shown to participants:
Help us make this tool better for you to use.

Response:

10. Please give any other comments or suggestions below.

Response type: Free-text response
Required: No

Response:
